# Polymorphisms of the lipoprotein lipase gene as genetic markers for stroke in colombian population: a case control study

**Published:** 2016-12-30

**Authors:** Leydi Carolina Velásquez Pereira, Clara Inés Vargas Castellanos, Federico Arturo Silva Sieger

**Affiliations:** 1 Departamento de Ciencias Básicas, Facultad de Salud, Universidad Industrial de Santander, Bucaramenga, Colombia.; 2 Grupo de Ciencias Neurovasculares. Fundación Cardiovascular de Colombia, Bucaramanga, Colombia.

**Keywords:** Coronary Disease, Lipoprotein lipase, Genotype, Myocardial Infarction, Polymorphism Genetic, Lipoprotein Lipase, Polymorphism Single Nucleotide, Risk, Colombia

## Abstract

**Objective:**

To analyze if there is an association between the presence of polymorphisms in the *LPL* gene (rs320, rs285 and rs328) with development of acute ischemic stroke in Colombian population.

**Methods:**

In a case control design, 133 acute ischemic stroke patients (clinical diagnosis and x-ray CT) and 269 subjects without stroke as controls were studied. PCR -RFLP technique was used to detect rs320, rs285 and rs328 polymorphisms in the LPL gene.

**Results:**

In the present research was not found any association between any of the LPL gene polymorphism and acute ischemic stroke in the population studied; the allele and genotypic frequencies of the studied polymorphisms were similar in cases and controls and followed the Hardy-Weinberg equilibrium. The study was approved by the IRB and each subject signed the informed consent.

**Conclusion:**

LPL gene polymorphisms are not genetic markers for the development of stroke in the Colombian sample used.

## Resumen

**Objetivo:**

Determinar la asociación entre los polimorfismos en el gen *LPL* (rs320, rs285 y rs328), y la enfermedad cerebrovascular isquémica aguda en una muestra de población colombiana.

**Métodos:**

A partir de un diseño de casos y controles, se estudiaron 133 casos con enfermedad cerebrovascular isquémica aguda (diagnóstico clínico y TAC), y 269 controles sin enfermedad cerebrovascular. Se examinó los polimorfismos rs320, rs285 y rs328 en el gen *LPL* con la técnica PCR-RFLP.

**Resultados:**

En el presente estudio no se encontró asociación entre rs320, rs285 y rs328 con la enfermedad cerebrovascular isquémica aguda en la muestra analizada; siendo las frecuencias alélicas y genotípicas de los polimorfismos similares entre casos y controles, y se encontró en equilibrio de Hardy-Weinberg. El estudio fue avalado por el comité de ética de las instituciones vinculadas y todos los pacientes dieron consentimiento informado.

**Conclusión:**

Los polimorfismos en el gen de la LPL no tienen utilidad como marcadores genéticos asociados con la presentación de la enfermedad cerebrovascular isquémica aguda en la muestra analizada.

## Introduction

Cerebrovascular diseases are characterized by a neurologic deficit, due to a focal vascular lesion in the central nervous system, it includes cerebral infarction, intracranial hemorrhage and subarachnoid hemorrhage ^1^. Various factors have been associated with increased stroke risk, blood pressure, lipid metabolism imbalance, diabetes, smoking, diet, obesity and insufficient physical activity. However, these factors do not explain completely disease development. Therefore, it is required to extend the studies about other risk factors or emerging factors, which could explain differences in stroke onset in different populations, and possible population susceptibility. In the latter case, genetic factor may play an important role as potential risk factors.

Lipoprotein lipase (LPL) catalyzes triglycerides (TG) hydrolysis from circulating chylomicrons and very low-density lipoproteins (VLDL), producing cholesterol rich lipoprotein remnants and components of high-density lipoprotein (HDL). The *LPL* gene located in region 8p22, spanning about 30 Kilobases (kb) and containing 10 exons, 9 of them codify for a protein of 475 amino acids. Genetic studies have revealed almost 100 mutations and single nucleotide polymorphisms in *lipoprotein lipase* gene ^2^ show that variations in this protein can affect the development of atherosclerotic plaque, and increase the risk of stroke. Some polymorphism in the *lipoprotein lipase* gene may play a role as risk or protection factors for stroke onset. For the first case, rs320 (8:19961566) result of thymine (T) to guanine (G) transition in the intron 8, and rs285 (8:19957678) result of cytosine (C) to thymine (T) transition in the intron 6, are associated with elevated TG and HDL ^3^ ^,^ ^4^. Contrariwise, rs328 (8:19962213) result of cytosine (C) to guanine (G) transversion in exon 9, it is identified as protective factor for stroke development, because it increases the proteolytic activity of the protein, increasing HDL levels and decreasing TG ^5^.

These polymorphisms have been studied in several populations, and it show discrepancies regarding their influence on stroke onset, it depends on the ethnic group and patient origin. The analysis of *LPL* polymorphisms rs320, rs285 and rs328 and the study of their distribution among Colombian population becomes the first study in Colombia to evaluate their association with stroke development. We hypothesize that polymorphisms (rs320, rs285 and rs328) located in the *lipoprotein lipas*e gene acts as exposure markers for development of stroke in Colombian population.

## Materials and Methods

### Design and population

Case control study. One hundred and thirty three patients, both genders, older than 18 years old, with ischemic stroke with diagnosis supported by computer tomography were recruited from medical centers in Cali (Fundación Valle de Lili), Bogota (Santa Clara Hospital), Bucaramanga (Fundación Cardiovascular de Colombia) and Medellin (San Vicente de Paul Hospital), between 2003 and 2007. As a control group, 269 healthy individuals, both genders, older than 18 years old who visited as visitors during the same period, the medical centers were recruited. They did not have any history or family history of stroke. The population was recruited in the frame of a previous study that analyzed risk factors for ischemic stroke in Colombian population ^6^.

The study was approved by ethical committee of the institutions involve in the study, patients signed an inform consent to participate in the study. 

We excluded patients who recent underwent under a surgery (previous 30 days), with cancer, hematological, hepatic or renal (creatinine higher than 1.5 mg) diseases, patients taking anti-inflammatory drugs (except ASA in doses as antiplatelet 75 to 300 mg/dL), pregnant woman and patient with cardio embolic diseases. 

Sociodemographic information such as age, gender and exposure factor as lipid profile (total cholesterol, triglycerides, HDL cholesterol, VLDL cholesterol, and LDL cholesterol), lipid-lowering drugs and genotyping were analyzed.

### Sample size calculation

Sample size calculation was estimated taking into account the frequency of the most frequent allele with lowest prevalence for the polymorphisms rs320, rs285 y rs328 in cases, previously reported in studies. EPIDAT 4^®^ software was used.

Nicklas *et al*. ^7^, found in American population that the frequency with the lowest prevalence for rs320 polymorphism was G allele (39%), for rs285 T allele (39%) and for rs328 G allele (15%), the latest prevalence was used to calculate sample size, because was the lowest from the three analyzed polymorphisms. For sample size calculation we took into account CI= 95%, power= 86%, OR for rs320 and rs285= 2.0 and for rs328= 0.5, case/control ratio= 1:1 (Table 1). Furthermore, sample size adjustment was performed for data loss. 

**Table 1:** Sample size and power calculation.

Polymorphisms	Allele	Frequency* (%)	OR	Sample size (Cases/controls)	Power (%)
rs320	G	39	2.0	182/182	86.0
				133/269	89.3
rs285	T	49	2.0	162/162	86.0
				133/269	92.5
rs328	G	15	0.5	244/244	86.0
				133/269	78.8

*Allele frequency taking from reference ^7^

### DNA analysis

DNA was extracted from the buffy coat from each patient that was stored at -70° C, using phenol-chloroform technique. The three polymorphisms of *LPL* were determinate using polymerase chain reaction-restriction fragments length polymorphisms (PCR-RFLP)

### Amplification of genomic DNA

Genomic DNA (13uL) was mixed with 200 µmol from each primer, 50 µmol/L from each of the four nucleotides, 2.5 mM de MgCl_2_ y 1 U de Taq polymerase (Promega^®)^. The final volume of the PCR mix was 25 µL. Each polymorphism had its own amplification protocol using different annealing temperatures. General amplification protocol was initial denaturation 94° C for 5 minutes, followed by 30 cycles; at 94° C for 5 minutes, annealing (rs320 55° C, rs285 57° C and rs328 60° C) for 1 minute and elongation 77° C for 10 minutes, and a final elongation at 77° C for 10 minutes. Oligonucleotides primers for PCR amplification of the three-studied polymorphisms, enzymes for digestion, electrophoresis gel and resulting fragments are described in Table 2. 

**Table 2:** Oligonucleotide primers for PCR amplification of the three studied polymorphisms of the LPL gene, digestion enzymes, resulting fragments. Electrophoresis was done using 2% agarose gel, 80V for 45 minutes and 37° C y 16° C.

Primers (5^'^->3^')^	Enzymes and palindromic sequences	Resulting fragments^*^	Allele
rs328	4 U MnlI	315 and 20	C
5^'^-CTGATTCTGATGTGGCCTGA-3^'^	5^'^...CCTC (N)_7_^...3^'^		
5^'^-CATGAAGCTGCCTCCCTTAG-3^'^	3^'^...GGAG (N)_6_^...5^'^	256, 59 and 20	G
rs320	4 U HindIII	140 and 210	T
5^'^-GATGCTACCTGGATAATCAAAG-3^'^	5^'^-A^∇^AGCTT-3^'^		
5^'^-CTTCAGCTAGACATTGCTAGTGT-3^'^	5^'^-TTCGA_∆_A-3^'^	350	G
rs285	4 U PvuII	431	C
5^'^-ATCAGGCAATGCGTATGAGGTAA-3^'^	5^'^-CAG^∇^CTG-3^'^		
5^'^-GAGACACAGATCTCTTAAGAC-3^'^	5^'^-GTC_∆_GAC -3^'^	222 and 209	T

^*^DNA 50 bp marker. All data in base pair

### Digestion and electrophoresis

Digestion of PCR product was performed by addition of 5 µL respective restriction enzyme and aqueous bathed at 37° C overnight. Restriction enzymes used were HindIII (New England biolabs^®)^, PvuII (New England biolabs^®)^, MnlI (New England biolabs^®)^ for the mutations rs320, rs285 y rs328, respectively. The resulting fragments were resolved by electrophoresis (80V, 45 minutes) on 2% agarose gel, stained with ethidium bromide and visualized on UV light.

### Statistical analysis

A duplicated database was constructed, its content was verified using validate tool from Epi Info^®^, to estimate mean, standard deviation, frequencies and percentages from the variables. Categorical variables were analyzed by chi-square test and continuous variables by students test. Odds ratios 95% CI and *p*-values were calculated as a measure of the association of each LPL genotype and phenotype with stroke. The probability of having a specific allele is defined as the frequency of carrier subjects over the frequencies of non-carriers subjects. The odds ratio for stroke is the probability of the group of patients having the allele associated with the disease onset versus the control group. A *p*-value of <0.05 was considered statistically significant for all test. Statistical analysis were performed by Stata 12 ^®^ y SPSS 19 ^®^.

Allele frequencies in cases and controls were evaluated by Hardy-Weinberg equilibrium test. Equilibrium state occurs when random mating has occurred. Equilibrium state indicates that the studied population is homogeneous regarding to the different alleles frequency and distribution, and it is not a mixture of subpopulations with different frequencies and allele distribution for the gene of interest. In the latter case (lack of equilibrium), if the subpopulation had a different risk of having stroke, cases and controls would not be comparable. Linkage disequilibrium is the tendency of the alleles of two separate but linked locus to occur more frequently that might be expected by chance. We evaluated it by estimating the observed frequencies of the haplotypes, i.e., all possible combinations of alleles at the loci rs320, rs285 y rs328. Population genetic analysis was performed with SNPStats y Arlequin 3.5.1.2^®^. 

## Results

### Sociodemographic and biochemical characteristics

We included 402 individuals, 133 cases y 269 controls. Mean age was 69 years in the cases group and 64 years in the controls, distributed equally between males and females. The clinical and biochemical characteristics of the study population are given in Table 3. Triglycerides, LDL cholesterol and VLDL cholesterol levels were similar between both study populations. The age of the stroke patients was significantly higher than the controls. Patients with stroke had significantly lower total cholesterol and HDL concentrations than healthy individuals. The percentage of controls who were taking lipid-lowering drugs was higher than the cases; however, we did not find significant differences. 

**Table 3:** Sociodemographic and biochemical characteristics.

Variables	Cases	Controls	*p*
	(n=133)	(n=269)	
Age (years-range)	69 (27-93)	64 (26-92)	0.00*
Female	58	136	0.19
Triglycerides (mg/dL)	162.48 ± 93.95	156.40± 68.46	0.46
Total cholesterol (mg/dL)	201.35 ± 52.88	212 ± 43.88	0.03*
LDL cholesterol (mg/dL)	129.43 ± 44.42	130.61± 41.11	0.79
HDL cholesterol (mg/dL)	39.42 ± 12.32	50.17 ± 12.66	0.00*
VLDL cholesterol	32.81 ± 18.78	32.59 ± 17.87	0.91
Lipid lowering drugs (%)	16 (12.0)	45 (16.7)	0.22

Data are presented as means ± SDs or number of subjects with total percentage in brackets. *p*-values were calculated using t-student and chi-square test.

*p-value <0.05

A significant difference between both groups was found for age, total cholesterol and HDL levels. Therefore, we performed a logistic regression analysis, it revealed an OR= 1.04 (CI 95%: 1.03-1.05) for age, total cholesterol OR= 1.00 (CI 95%: 0.99-1.00) and HDL cholesterol OR= 0.94 (CI 95%: 0.93-0.95). 

### Genotyping

**Figure 1:**
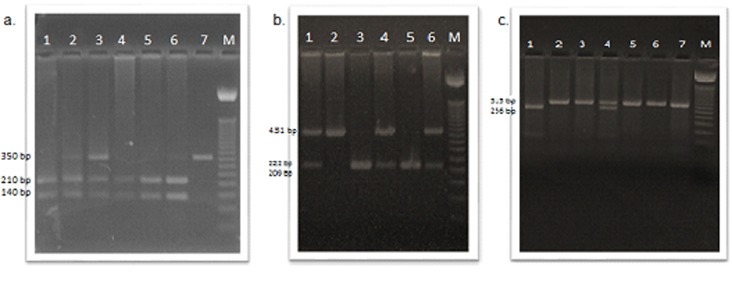
Genotyping for rs320, rs285 y rs328 polymorphisms in *lipoprotein lipase* gene. Restriction fragments length polymorphisms (RFLP) on 2% agarose gel. a. rs320; lane 1, 4, 5, 6, TT genotype; lane 2, 3, GT genotype; lane 7, GG genotype; b. rs285; lane 1, 4, 6, CT genotype; lane 2, CC genotype; lane 3,5, TT genotype. c. rs328; lane 1, GG genotype; lane 2, 3, 5, 6, 7, CC genotype; lane 4 CG genotype; M, DNA 50 bp marker*.*

Polymorphisms allowed making the allele assignation in each sample for each polymorphism (Fig. 1).

### Genotype and allele frequencies

Genotype and allele frequencies for rs320, rs285 y rs328, did not differ significantly between controls and cases. For rs320 polymorphism, the most frequent genotype was TT in both study population 51, and 52% respectively. The most common genotype between cases and controls for rs285 was the CT with a frequency of 45% and 48% respectively. For rs328 polymorphism, G allele had a low frequency in both groups, 9% and 7% respectively, and GG genotype had the lowest frequency in both populations, with a value of 1% (Table 4).

**Table 4:** Genotypic and allele frequency for rs320, rs285 y rs328 polymorphisms.

Polymorphism	Cases (n= 133)*	Controls (n= 269)*	*p*
Genotype rs320			
GG	18 (13.5)	29 (11.0)	0.42
GT	46 (34.6)	103 (38.0)	0.47
TT	69 (51.9)	137 (51.0)	0.86
Allele rs320 T†	69.0	70.0	0.79
Genotype rs285			
CC	43 (32.0)	73 (27.0)	0.28
CT	64(48.0)	121 (45.0)	0.55
TT	26 (20.0)	75 (28.0)	0.07
Allele rs285 T†	44.0	50.0	0.07
Genotype rs328			
CC	110 (82.7)	232 (86.0)	0.35
CG	22 (16.5)	34 (13.0)	0.29
GG	1 (0.8)	3 (1.0)	0.73
Allele rs328 G†	9.0	7.0	0.43

*n (%)

† %

*p*-values were calculated using chi-square test.

The population was in Hardy-Weinberg equilibrium (*p*= 0.05), for rs285 and rs328 polymorphism in cases and controls. In contrast, rs320 polymorphism was in Hardy-Weinberg equilibrium in the controls, but no in the cases. However, we applied the Bonferroni correction, we adjusted the significance value to *p*= 0.02, this new value allowed that the population reaches Hardy-Weinberg equilibrium.

### Haplotype analysis

Results showed 8 possible haplotypes. We did not find significant differences in the haplotype frequencies distribution between the two groups. Haplotype frequencies for each haplotype are shown in Table 5. The most common haplotype was TCC and the less frequent haplotype was GTG. 

**Table 5:** Haplotype frequencies of the LPL gene (rs328, rs285 and rs320) in stroke patients and controls

Haplotype	Cases	Controls	*p*
	% (n= 133)	% (n= 269)	
GCC	2.03	2.41	0.74
GCG	2.62	2.41	0.86
GTC	4.94	4.82	0.94
GTG	0.87	0.60	0.68
TCC	18.02	18.37	0.91
TCG	5.52	4.22	0.43
TTC	59.01	59.34	0.93
TTG	6.98	7.83	0.67

*p*-values were calculated using chi-square test

Probability value for markers without gametic association was evaluated using chi-square test. We found for all pairs of loci, *p*-values lower than 0.05, it shows that there is a gametic association between the loci.

### Association between polymorphisms and stroke

In order to study a possible association between the polymorphisms and stroke develop, we calculate OR value for each of the inheritance patterns. We did not find association by evaluating OR between polymorphisms and stroke development (Table 6). 

**Table 6:** Association for each inheritance patter with stroke

Polymorphism	Model	Genotype	OR	IC 95%	*p*
Rs320	Codominant	TT	1.00		0.73
		GT	1.14	0.72- 1.80	
		GG	0.87	0.45- 1.70	
	Dominant	TT	1.00		0.78
		GT-GG	1.06	0.70- 1.62	
	Recessive	TT-GT	1.00		0.56
		GG	0.82	0.43- 1.56	
Rs285	Codominant	CC	1.00		0.14
		CT	1.15	0.70- 1.89	
		TT	1.78	0.98- 3.22	
	Dominant	CC	1.00		0.22
		CT-TT	1.34	0.84- 2.12	
	Recessive	CC-CT	1.00		0.06
		TT	1.63	0.98- 2.72	
Rs328	Codominant	CC	1.00		0.59
		CG	0.77	0.43- 1.40	
		GG	1.87	0.19-18.58	
	Dominant	CC	1.00		0.50
		CG-GG	0.82	0.46- 1.46	
	Recessive	CC-CG	1.00		>0.64
		GG	0.94	0.20-19.26	

## Discussion

Data shows no significant association between *lipoprotein lipase* gene polymorphisms rs320, rs285, rs328 and stroke in the Colombian simple analyzed; suggesting that this polymorphism cannot be used as genetic markers to predict risk for stroke development. This is the first study done in Colombia searching for genetic marker that may have an association with stroke in Latin America to date, only brazil have published this kind of studies, finding an association between rs320, rs285, rs328 with stroke development ^5^ ^,^ ^8^ ^,^ ^9^. However, in other populations the results changed.

Allele frequencies for rs320, rs285 y rs328 in our study were nearly to those found in in the dbSNP database for European population and genetic studies done in different ethnic groups ^7^ ^,^ ^10^ ^,^ ^11^. Additionally, the allele frequencies of the three genotypes did not differ from the expected frequencies according to the Hardy-Weinberg equilibrium from both the control group and stroke patients. It shows that there are no significant differences between the prevalence of the corresponding genotypes to the polymorphisms in the lipoprotein lipase gene, suggesting a lack of association of these polymorphisms with stroke development.

We compared lipid profile results with reference values established by the National Cholesterol Education Program, and we found that total cholesterol and triglycerides levels were in the borderline high in both groups. LDL levels in cases were optimal. However, for controls were borderline high. Additionally, we performed a logistic regression, and we used the variables, as a result, there were found a significant difference among cases and controls. We found that age acts as a risk factor for stroke development. Moreover, HDL cholesterol acts as a protective factor for stroke development, similar to previous reports ^12^. We did not evaluated the LPL polymorphisms association with altered lipid profile since previous studies failed to do so or had led to inconsistent results ^13^.

Several studies have evaluated rs320, rs285 y rs328 polymorphisms in the lipoprotein lipase gene and their association with stroke. Rs328 have been widely analyzed, however, results regarding its lipolytic activity and effect as protective factor differ according the population, in some, and they found an increase in lipoprotein lipase activity and in others a 45% increase of the normal lipolytic activity. They found a protective effect in the following populations; French ^14^, Japanese ^15^, Chinese ^16^, Finn ^17^, English ^18^, Swedish ^19^, Brazilian ^5^ ^,^ ^8^, Arabic ^20^, Dutch ^21^, but no in Greeks ^22^ nor American ^7^.

Studies in population from United States ^7^, France ^14^, Wales ^11^, India ^23^, China ^16^, Japan ^15^, Brazil ^5^, and Tunisia ^24^ y Germany ^25^ found that rs320 polymorphism alters lipid levels and acts as a predisposition marker for stroke development. Nevertheless, in Russia ^26^, Saudi Arabia ^20^ and Italy ^27^ they did not find any association.

rs285 have been associated with high triglycerides and low HDL cholesterol levels and an increase in the severity of coronary heart disease in diabetes mellitus II patients. This was found in people from China ^16^, United States ^7^ ^,^ ^28^, Brazil ^5^, Australia ^29^, France ^14^ and Japan ^15^. Whereas in Macedonia ^30^ and Saudi Arabia ^20^ they did not find any difference regarding the distribution of the polymorphism in cases and controls.

rs320, rs285 y rs328 polymorphisms in *lipoprotein lipase* were selected because they are the most common polymorphisms reported in literature, and they have been found to be associated with alterations in lipid profile. We studied rs320 y rs285 polymorphism, which are located in intronic regions of the gene (8 and 6, respectively); because it has been demonstrated, that certain intronic region contains host elements that regulate gene transcription and translation. Moreover, they act as markers for functional mutations and alter amino acids sequence ^3^. In this study, we did not find any association between rs320 and rs285 with stroke, maybe because these mutations are located in loci, in which, it is unlikely that it plays a functional role. Additionally, rs328 might be involve in cardiovascular haemostasis, and might be affected by other genetic, environmental or both factors ^22^. Moreover, stroke is a multifactorial disease; therefore, it is possible that several genes influence the disease outcome, in other words, a result of complex interactions between genetic predisposition and environment. 

Two limitations could be pointed out in relation to our study: the first was the relatively small sample size. It is possible that the association between the markers and stroke it is low and the sample size is not big enough to detect this association or there are other exposure markers for stroke development. And the second was the statistical power for the results of rs320 and rs285 polymorphisms have a statistical value higher than 86%. In contrast, the statistical power for rs328 was lower than 86%, therefore the association analysis for this polymorphism lacks of enough power. However, we provide prevalence information of this polymorphism in our population.

Genetic markers analysis for stroke is a simple and accurate tool to predict disease risk, which provides information that can be used to improve the distribution of health budget, identify people with high risk and recruit them through the health system. It allows the identification of associated risk factor, monitor trends trough the time. Providing a base to design and implement interventions, monitor and evaluate the efficacy of these interventions, creation of research networks, determinate country priorities to prevent and treat stroke in the national plans context to prevent and control chronic diseases and compare data between and within populations. However, the current study shows that the difference in the distribution of rs320, rs285 y rs328 polymorphisms in *lipoprotein lipase* gene, in patients with stroke and healthy population, do not act as statistically significant risk markers for stroke development in the our population. 

Future studies should evaluate other SNPs that may influence other risk factors for stroke, such as obesity, diabetes, high blood pressure, taking into account that some environmental factors may affect disease development. With the support of molecular, biological and genetic techniques allows elucidate etiophatogenic mechanisms for stroke. 
